# Discoloration of Gold Fillings

**Published:** 1893-08

**Authors:** 


ARTICLE II.

### DISCOLORATION OF GOLD FILLINGS.

This has puzzled many of our best dentists.

"From amalgam on our polisher?" Then why should one filling be stained and others remain bright? Yes, mercury will do it, whether conveyed on an instrument or in ptyalism; but they become tarnished when no amalgam has been used or administered.

"Impure gold?" But with gold from the same book, some will remain bright and some be tarnished.

"Filthiness of the mouth?" Then why should some of our cleanest patients complain, and others who never brush their teeth be exempt?

Let me illustrate the facts and try to show the cause by an anecdote.

A gentleman came into my office with his son, for whom I had filled several teeth with gold, saying:

"Dr. Welch, just look at my boy's teeth you filled. What can you say of such work as that? I sent him to you as being a specially good dentist, and I paid you a specially good price. Now look at the result. Nearly half the fillings have turned so dark they are a disgrace to you and to the boy."

"Not half, Professor Wright. Let me see, only four of the twelve; but this is quite enough. These four do look as though they had been in the fire; or at least they are tarnished as though composed of brass or some other base metal. But, Professor, why should they not all look alike? These two apposing fillings in the right lateral and cuspid, and their opposites on the left side, have turned quite dark, and all of the other fillings remain bright, though the twelve fillings were made at the same time from the same book of gold; even these in the centrals are unaffected. And look here, Professor; on the right lower teeth are four filled with my gold and platina alloy, all untarnished but one. This one, covering the whole buccal side of the second molar, and dipping down quite below the gum, is as black as my hat, though it is my boast that my gold and platina filling seldom tarnishes. Now you are a teacher of chemistry of some celebrity, explain this difference."

"It is not for me, sir, to explain, but for you. I believe you have filled these teeth with poor material."

"But I aver most solemnly that all this gold is of the same quality, taken from the same book. If the material is poor in one it is poor in all. Besides, you see the same discrepancy in the appearance of those filled with my alloy. As a chemist, please explain the cause and the exceptions; for this is not the first instance of the kind. Many dentists are inquiring why this is thus, as an occasional phenomena, and erratic when it does appear, coloring one filling and unaffecting others."

"Well, come, if you have any *facts* you think will lift the blame from your shoulders, let me have them."

"Well, Professor, here is a piece of litmus paper. Suppose you place it against the surface of the fillings next to the gum."

Contact with the unaffected fillings produced no discoloration of the paper, but its pressure against the discolored fillings proved the presence of acid.

"But this acidity is only slight," said he; it is not sufficient to produce such an effect as we see here."

"No;" we replied; "but put it against the discolored fillings again. Now press the gum so as to bring out what may be just under its edge."

Immediately the litmus paper was turned decidedly red.

"That's wonderful," said he; "and I find no such effect in pressing it ever so firmly against the margins of the gum elsewhere. What kind of acid can it be?

"Whatever kind it is," we replied, "it is undoubtedly the same that sometimes discolors the teeth themselves next to the gums, and sometimes actually disintegrates its surface. But let me surprise you again, though you have been a professor of chemistry for years. This decidedly strong action on these teeth and fillings is the nascent combination of an acid with an alkali. If you care to demonstrate the fact, you will find it the union of nitric acid with ammonia. In the little pockets under the gum of these affected teeth is found from the decomposition of nitrogenous food, nitrate of ammonia. Why this sudden and unusual combination should show itself in some mouths and not in others, and with such virulence as to discolor the best gold, or rapidly to disintegrate the surface of the neck of a tooth, and this in one portion of the mouth, and not in another, and why it should show itself in any mouth, and then in such an isolated and erratic manner, I cannot divine. Before we further discuss its philosophy and chemistry, suppose you make it a study for a time. But while you are becoming satisfied, be sure you require your son to keep his teeth thoroughly clean, using good soap and a little chalk."

He became satisfied the fault was in the condition of the mouth, and not in the quality of the gold.—EDITORIAL in *Items of Interest.*

---

ARTICLE III.

